# Psychological Factors of Long-Term Dietary and Physical Activity Adherence among Chinese Adults with Overweight and Obesity in a Community-Based Lifestyle Modification Program: A Mixed-Method Study

**DOI:** 10.3390/nu12051379

**Published:** 2020-05-12

**Authors:** Alice W. Y. Leung, Ruth S. M. Chan, Mandy M. M. Sea, Jean Woo

**Affiliations:** 1 The Nethersole School of Nursing, Faculty of Medicine, The Chinese University of Hong Kong, Hong Kong, China; 2 Department of Medicine and Therapeutics, Faculty of Medicine, The Chinese University of Hong Kong, Hong Kong, China; ruthchansm@cuhk.edu.hk (R.S.M.C.); jeanwoowong@cuhk.edu.hk (J.W.); 3 Centre for Nutritional Studies, Faculty of Medicine, The Chinese University of Hong Kong, Hong Kong, China; mandysea@cuhk.edu.hk

**Keywords:** diet, physical activity, psychological factors, adherence, obesity management, adults, healthy lifestyle, mixed-method study

## Abstract

There is a paucity of research on factors influencing long-term adherence to lifestyle modification. We conducted a mixed-method study to explore the psychological factors of dietary and physical activity (PA) adherence among Chinese adults with overweight and obesity at 10 months after enrollment of a community-based lifestyle modification program in Hong Kong. We recruited Chinese adults newly enrolled in a culturally adapted lifestyle modification program and followed them for 10 months. For the quantitative study, primary outcomes were dietary and PA adherence scores while secondary outcomes included knowledge, self-efficacy, motivation and stage of change. For the qualitative study, data were collected using semi-structured interviews and observation. A total of 140 participants completed the 10-month follow-up. They reported moderate level of dietary adherence but low level of PA adherence at 10 months. Multivariable regression analyses revealed that greater improvement in nutrition knowledge and diet stage of change predicted higher dietary adherence while greater improvement in PA self-efficacy and PA stage of change predicted higher PA adherence. Qualitative data on 26 participants suggest that participants’ knowledge and self-efficacy but not motivation were enhanced during the program. The findings of this study enhanced our understanding on factors influencing long-term adherence to lifestyle changes.

## 1. Introduction

Regardless of the significant efforts made to address the obesity epidemic, obesity remains a major public health concern [1]. There has been substantial evidence for successful weight loss based on various intervention methods including diet, physical activity (PA) and medications. Nevertheless, many regained the lost weight within one year regardless of the methods used [2,3,4]. Very often, weight rebound is attributed to the failure to maintain healthy eating and PA behaviors over time [5,6].

A comprehensive lifestyle modification program (LMP) typically comprises a combination of diet, PA and behavioral approach, is recognized as the most cost-effective and sustainable option for weight loss [7]. However, the long-term efficacy of LMPs is not well defined, due to high dropout rates and limited long-term studies [7,8]. Identifying factors of long-term dietary and PA adherence can enhance our understanding on the facilitators for sustaining long term diet and PA behavioral changes, which have the potential to inform the development of evidence-based, sustainable obesity management strategies.

A review of the literature concerning factors influencing adherence to LMP found a wide range of potential factors (grouped in four categories: psychosocial, socio-demographic, behavioral and physical factors) but the evidence for factors of behavior specific adherence is scarce and long-term prospective studies are largely lacking [9]. More recently, there has been budding interest in factors associated with behavior specific adherence to weight loss or weight maintenance plan. In a N-of-1 study using daily ecological momentary assessment, Kwasnicka et al. identified motivation, satisfaction with outcomes, self-regulation, habit and stable environment as predictors of self-reported dietary and PA adherence over 6 months among eight British adults who had intentionally lost >5% body weight in the previous year [10]. Cruwys et al. examined the psychosocial predictors of dietary adherence among a diverse sample of Australian adults who were following five different restrictive dietary patterns: vegan, vegetarian, paleo, gluten-free, and weight-loss. The findings suggested that self-efficacy, social identification and motivation were predictors of dietary adherence [11]. Fitzpatrick et al. found that age, race/ethnicity, BMI at screening, vitality, encouragement from family and friends were predictors of adherence to dietary and PA recommendations one year after the intensive weight loss phase among participants of the Weight Loss Maintenance and PREMIER trials [12].

However, there is still a large gap on factors influencing long-term adherence to lifestyle changes. To address the current literature gap, we conducted a cohort study, in which participants of a culturally adapted LMP were being followed from the beginning of the program until 10 months later. We developed program-specific adherence scores to indicate dietary and PA adherence. In a previous publication, participants reported high dietary adherence but low PA adherence in the early weight loss phase. Several psychological predictors of dietary and PA adherence outcomes were identified. Diet self-efficacy (baseline) and nutrition knowledge (one-month change) were independent predictors of dietary adherence while autonomous PA motivation (baseline) and PA self-efficacy (both baseline and one-month change) were independent predictors of PA adherence [13].

Based on the previous report on predictors of adherence in early weight loss phase, the aim of this mixed-method study was to explore the psychological factors of dietary and PA adherence at 10 months after enrollment, which corresponded to the early weight maintenance phase in the majority of participants. In the quantitative study, we hypothesized that greater increase in knowledge, self-efficacy, motivation, or stage of change would predict higher dietary and PA adherence at 10 months. Furthermore, the following two research questions guided the qualitative study:

1. How did participants perceive the influence of the program on their knowledge, self-efficacy and motivation?
2. During consultations, how did the registered dietitians (RDs)/nutritionists/fitness specialist enhance the knowledge, self-efficacy and motivation of participants?

## 2. Materials and Methods

### 2.1. Design

This study adopted a sequential explanatory strategy using a mixed methods approach, collecting both quantitative and qualitative data. The selection of the sequential explanation strategy was guided by the criteria proposed by Creswell [14]. Priority was given to quantitative data collection and qualitative data collection came after preliminary analysis of quantitative study. Integration of the data occurs during data interpretation [14]. In the quantitative study, we analyzed the baseline and 10-month follow-up data of the cohort study. The qualitative findings were used to assist in explaining and interpreting the findings of the quantitative study. In addition, it could help to explain any unexpected results arising in the quantitative study [14].

### 2.2. The Program

The culturally adapted LMP has been established under the Center for Nutritional Studies (CNS) of the study institution since the early 2000s. The program, also known as CNSLMP, is a self-financing weight management program open for public enrolment. The details of the program and its theoretical foundations have been published previously [15,16]. CNSLMP provides individual dietary and PA counselling using behavioral approaches. During the program, clients are followed by the same RDs/nutritionists and a fitness specialist. In the first consultation, RDs/nutritionists conduct comprehensive health and dietary assessments, help clients set realistic weight loss goals and create tailored diet plans. Clients are advised to have weekly follow-up sessions for the first three months and monthly sessions thereafter. The program length varies among individuals considering clients’ initial weight status, their weight loss goals, as well as their weight loss progress [15,16]. The regular consultations cover topics on balanced diet, food substitution, food exchange lists, food labels, eating out tips, cooking methods and active lifestyle. Clients are required to complete daily food and PA diaries for the purpose of self-monitoring [15,16]. Other than the regular consultations, at least one PA consultation with the fitness specialist is arranged for each client. The fitness specialist assesses their fitness level and prescribes individualized PA plans in accordance with the ACSM’s Guidelines for Exercise Testing and Prescriptions [17]. Upon completion of the weight loss phase, participants are offered an annual advance membership on CNS, which included four weight maintenance consultations and was worth HKD$800–$1000 (~103–129 USD) during the study period. Subscription to this membership is encouraged but not mandatory. Nevertheless, recommendations on weight maintenance diets and PA were given by RDs/nutritionists upon completion of the weight loss phase. Previous evaluative studies supported the effectiveness of CNSLMP in weight loss maintenance. In a cross-sectional telephone survey on 602 completers with average weight loss of 16% upon completion of the program, 61.1% were able to maintain weight loss (<25% weight gain) after 2.7 ± 0.9 years [18]. In a 12-month randomized control trial with non-alcoholic fatty liver disease patients, the proportion of patients in the lifestyle intervention group (77%) with sustained weight lost >3% was significantly greater than in the usual care control group (30%) at 12 months [19].

### 2.3. Participants

A convenience sampling approach was adopted during recruitment. The participants were recruited from the two centers run by the CNS, one located in a residential area and another located in a business district. All newly enrolled Chinese clients who were aged 18–65 years and overweight or obese (BMI > 23 kg/m^2^ based on BMI cut-off points for Asian population [20]) were recruited to the study. Those with histories of psychiatric disorders; taking weight loss medication; having a medical condition that would limit PA participation; and having other conditions that may interfere with the participation and ability to follow the intervention protocol (e.g., pregnancy, planning for pregnancy) were excluded. A total of 284 eligible clients were invited to take part in this study but 19 declined the invitation. Among the participants enrolled (n = 265) in the study, 205 completed the one-month follow-up, which represented the early weight-loss phase for the majority of participants [13]. In the present study, we conducted a follow-up study of these 205 participants at 10 months after enrollment of CNSLMP.

For the qualitative study, participants were drawn from the completers of the quantitative study. Participants were purposefully selected through a combination of maximum variation and convenience sampling strategies. Dietary and PA adherence at one-month follow-up [13] were used as the criteria for maximum variation sampling in order to capture the experience of participants across different levels of adherence. Consistent with the qualitative methodology, sample size was determined by the principle of saturation in which no additional data could be found in further interviews for a category [21]. Since PA consultations with the fitness specialist are rarely arranged for participants during weight maintenance in usual practice, an additional convenience sample of 10 Chinese clients with overweight and obesity was recruited for PA consultation observation.

### 2.4. Data Collection

#### 2.4.1. Quantitative Study

This study was conducted between April 2014 and August 2016. A research assistant approached eligible participants to brief them about the study details. After written consent was sought, the participants’ baseline characteristics including socio-demographic characteristics, general health and weight-loss history were collected. At baseline and 10-month follow-up, the Chinese versions of a program-specific knowledge questionnaire, Self-Rated Abilities for Health Practices Scale (SRAHP) [22], Treatment Self-Regulation Questionnaire (TSRQ) [23], Stage of Exercise Scale [24], and the 7-item International Physical Activity Questionnaire-Short Form (IPAQ-SF) [25] were administered. At 10 months, dietary and PA data were collected using 4-day food records and 7-day PA records, respectively. At both time points, body weight was measured to the nearest 0.1 kg using a Seca 220 measuring system (Seca 220; Seca, Hamburg, Germany). Upon the return of questionnaires, participants received HKD$50 cash (~6.5 USD) as incentive. Furthermore, a free annual advanced membership on CNS, which was worth HKD$800–$1000 (~103–129 USD) and included four free nutrition consultations, was offered upon completion of the study at 10 months. According to the Quarterly Report of Wage and Payroll Statistics published in December 2014, the median monthly average salary in Hong Kong was HKD$14,354 (~1852 USD) [26].

##### Outcome Measurements

Dietary adherence score was produced from scoring 4-day food records. Initially, the food records were analyzed by Food Processor software, version 8.0, by ESHA Research (Salem, OR, USA) to obtain the average daily total energy intake, % energy from carbohydrate, fat and protein, servings of fruits and vegetables. One score was allocated for fulfilling one criterion. The eight scoring criteria were described in detail in a previous publication [13]. The total dietary adherence score was computed by summing all the scores to maximum of 8 per day and 32 for four days.

The PA adherence score consists of two components: Program PA score and IPAQ PA score. Program PA score, which assesses the adherence to PA plan prescribed by the fitness specialist, was produced from scoring 7-day PA records. IPAQ PA score, which assesses change in PA from baseline to 10 months, was produced by scoring the 7-item IPAQ-SF [25]. One or two scores were allocated for fulfilling one criterion. The six scoring criteria have been described in detail in a previous publication [13]. The total PA adherence score were computed by summing all the scores to a maximum of 10.

The knowledge questionnaire, developed based on the contents of the program, comprises 33 items grouped in two scales: nutrition knowledge (22 items) and PA knowledge (11 items) scales. The nutrition knowledge scale covers topics on balanced diet, weight-loss diet, eating-out, cooking methods, and food exchanges. The PA knowledge scale covers the appropriate type, intensity, duration and frequency of PA. Of the 11 items, six items were extracted from the Chinese Physical Activity Questionnaire [27]. One score was allocated for each correct response. The nutrition and PA knowledge scores were computed by summing all correct answers. Seven RDs/nutritionists and the fitness specialist who were involved in delivering the program reviewed the questions to ensure their relevance to the contents of the program.

The Exercise and Nutrition subscales of the Chinese version of SRAHP were used to assess self-perceived ability to implement healthy eating and PA behaviors. Each subscale consists of 7 items rated on a scale of 0 (not at all) to 4 (completely). The Chinese version of SRAHP has shown high internal consistency (α = 0.92) and two-week test–retest reliability (0.78) among Taiwanese childbearing women [22].

The Chinese version of the 15-item TSRQ was adapted to assess autonomous and controlled motivation to eat a healthy diet and do regular PA. Each item was rated on a scale of 1 (not at all true) to 7 (very true). As the name of behavior in TSRQ can be slightly modified to accommodate different health behaviors, two sets of TSRQ were developed to assess diet and PA motivation. The Chinese version of TSRQ has demonstrated high internal consistency (α > 0.7) among Hong Kong Chinese athletes [23]. In addition, two self-developed items were added in TSRQ to examine motivation pertinent to body image.

The Chinese version of the Stage of Exercise scale, which uses a five-item, ordered-categorical scale, was adapted to assess diet and PA stage of change. This scale has shown high test–retest reliability (k = 0.85) among Taiwanese adults with overweight and obesity [24]. The wordings “physical activity” and “healthy diet” were used to replace ‘exercise’ in the original scale.

Prior to the commencement of the study, the knowledge questionnaire was pilot-tested in 27 clients for comprehension and question format while the other three questionnaires were pilot-tested in 29 clients with good internal consistency (α = 0.79–0.91) and reasonable to high test–retest reliability (r = 0.67–0.97).

#### 2.4.2. Qualitative Study

The principle researcher (A.W.Y.L.), who is a nutritionist and was not involved in delivering nutrition consultations, conducted all nonparticipant observation and in-depth semi-structured interviews held at CNS. The principle researcher observed the nutrition and PA consultations, lasting around 10–30 min, using the research question Q2 as a guide. Field notes were taken during observation. All participants were offered the opportunity to check the field notes after the observation, and any inappropriate content was deleted as requested. Thereafter, in-depth individual interviews, lasting around 30–45 min, were conducted in a separate room in the absence of RDs/nutritionists. All interviews were conducted with the support of an interview guide (Table S1). At the beginning of the study, two pilot interviews were conducted to refine the interview guide. The questions related to stage of change were deleted after pilot interviews as the concept appeared to be too academic and too difficult to be answered.

### 2.5. Ethical Considerations

Ethical approvals for the quantitative study and the qualitative study were obtained from the Joint Chinese University of Hong Kong—New Territories East Cluster Clinical Research Ethics Committee (CREC Ref. No.: 2013.623) and Survey and Behavioral Research Ethics Committee of the Chinese University of Hong Kong, respectively. All participants were briefed about the study details and their rights to withdraw with reference to an information sheet. Written consents were obtained from participants prior to data collection.

### 2.6. Data Analysis

#### 2.6.1. Quantitative Study

Data are presented using appropriate statistics: mean (standard deviation), median (interquartile range), and frequency (percentage) for normal-like, skewed, and categorical variables, respectively. Listwise deletion, which refers to the elimination of cases with missing data, was used in this study. Univariate comparison between completers and non-completers was performed by performing independent sample *t*-tests or chi-squared tests, whenever appropriate. Changes in psychological variables between baseline and the 10-month follow-up were compared using paired *t*-tests or Wilcoxon signed-rank tests, as appropriate. Linear regression analyses were performed for continuous outcomes (dietary or PA adherence scores). Multivariable regression models were created to identify independent predictors of adherence. Independent variables were changes in psychological variables from baseline to 10 months and baseline psychological variables. Baseline characteristics were entered as covariates. Covariates with a *p*-value < 0.2 in univariate analyses were selected as candidate variables for multivariable regression analyses. All statistical analyses were performed in STATA 13.0 software using two-sided significance tests with statistical significance set at 0.05.

#### 2.6.2. Qualitative Study

The tape recordings were transcribed verbatim in Cantonese. All field notes and transcriptions were coded and anonymous. The transcripts and field notes were imported in to NVIVO (Version 8. QSR International, Doncaster, Australia) for coding and analysis. Thematic analysis, which aims to identify themes from the data, was used for the purpose of data analysis [28]. The codes, themes and quotes were translated into English by the principle researcher who had experience in Cantonese–English translation. Another bilingual research assistant back-translated the English codes, themes and quotes into Cantonese to achieve semantic equivalence [29].

## 3. Results

### 3.1. Quantitative Findings

#### 3.1.1. Participants’ Characteristics

At baseline, the mean age and BMI of the 205 participants was 38.9 ± 10.5 and 28.4 ± 1.2, respectively. The majority of them were female (79.0%) and did not have smoking (88.3%) or drinking habits (68.8%). Slightly less than half of them were married (45.4%) and with one or more chronic diseases (43.9%), while slightly more than half of them had tertiary education or above (59.0%). Two thirds of the participants had a monthly family income of more than HKD$30,000 (~3825 USD). Around one third of them were professionals or associate professionals (31.5%). For weight loss history, the majority of them had weight that had gradually increased over the past 10 years (64.2%) and only 18.5% did not have any weight loss attempts before joining the program.

Of the 205 participants, 65 dropped out of the study at 10-month follow-up. Table 1 summarizes the comparison of baseline characteristics between completers and non-completers at 10-month follow-up. There were no significant differences in baseline characteristics except for gender and previous enrolment in the program. Of the 140 completers of the study, the mean duration of weight maintenance was 5.15 ± 3.15 months. All of them completed the weight loss phase before the 10-month follow-up, as confirmed by their RDs/nutritionists. Among the 140 completers, only 12 of them subscribed to the annual membership and completed a weight maintenance session at 10-month follow-up.

#### 3.1.2. Weight Outcomes

The mean weight loss of the 140 completers at 10 months was 5.80 ± 5.46 kg, corresponding to a 7.76 ± 6.69% loss of baseline body weight. Around two-thirds (64.7%) of participants had sustained 5% or more weight loss at 10 months, while around one-third (33.8%) of the participants had sustained 10% or more weight loss.

#### 3.1.3. Adherence Outcomes

The mean dietary adherence score at 10 months was 18.83 ± 6.05 (range: 0–32), indicating a moderate level of dietary adherence. On the other hand, the mean PA adherence score at 10 months was only 3.65 ± 2.13 (range: 0–10), indicating a low level of PA adherence. Weight loss was positively correlated with dietary (rho = 0.174 *p* = 0.043) and PA (rho = 0.278, *p* = 0.001) adherence scores (details not shown).

#### 3.1.4. Psychological Outcomes

The changes in psychological outcomes between baseline and 10 months were compared, and the results are presented in Table 2. At 10 months, participants had significantly higher nutrition and PA knowledge, diet and PA self-efficacies, controlled motivation for a healthy diet and PA, and higher diet and PA stages of change than baseline (*p* < 0.05). More profound increases were observed in nutrition knowledge and PA self-efficacy in comparison to other psychological outcomes (*p* < 0.001).

#### 3.1.5. Psychological Predictors of Dietary Adherence

A summary of regression analyses for dietary adherence score at 10 months is presented in Table 3. Univariate analyses revealed that increase in stage of change (β = 2.16, *p* = 0.036) was a significant predictor of 10-month dietary adherence score. In the multivariable model, increase in nutrition knowledge score (β = 0.57, *p* = 0.006) and increase in stage of change (β = 2.59, *p* = 0.020) remained significant after adjusting for baseline psychological variables and potential covariates. The baseline psychological predictors remained significant were nutrition knowledge (β = 0.50, *p* = 0.010) and stage of change (β = 3.95, *p* = 0.012).

#### 3.1.6. Psychological Predictors of PA Adherence

A summary of regression analyses for PA adherence score at 10 months is presented in Table 4. Univariate analyses revealed that increases in PA self-efficacy (β = 0.16, *p* < 0.001), autonomous motivation for PA (β = 0.08, *p* = 0.007) and stage of change (β = 0.97, *p* = 0.007) were significant predictors of 10-month PA adherence score. In the multivariable model adjusted for baseline psychological variables and potential covariates, increase in PA self-efficacy (β = 0.16, *p* < 0.001) and increase in stage of change (β = 0.83, *p* = 0.031) remained significant predictors of 10-month adherence score. Additionally, the baseline psychological predictor that remained significant was stage of change (β = 1.13, *p* = 0.030).

### 3.2. Qualitative Findings

A total of 27 out of the 140 participants in the quantitative study were contacted. One participant declined the interview. The remaining 26 participants agreed to take part in the nutrition consultation observation, followed by individual interviews. The baseline characteristics and program experience of the 26 participants are presented in Table 5. The interviews were conducted on average 11 months post weight loss phase and lasted for 22 min to 52 min. An additional 10 subjects with overweight and obesity were recruited on site for PA consultation observation. The 10 subjects were aged 21–60, with the majority being female (9/10). All of them were in the weight loss phase. Half of them had the PA consultation within one month after joining CNSLMP. Table 6 summarizes the themes, subthemes and codes generated during data analysis.

#### 3.2.1. Theme 1: RDs/Nutritionists or Fitness Specialist Enhanced Participants’ Knowledge

##### Nutrition Knowledge

During nutrition consultations, the majority of the time was spent on enhancing participants’ nutrition knowledge. Likewise, participants mentioned a variety of knowledge given by RDs/nutritionists and perceived that knowledge to be helpful for them to follow the prescribed diet plans. Four major types of knowledge were identified from the interviews and observation of diet consultations. These included individualized diet plans, portion counting, healthier food choices and strategies used to handle barriers.

*Individualized diet plans*

*“I followed her (Nutritionist’s) advice. During the program, she recommended different diet plans, for example, one for edema, one for big dinner and light lunch. I just follow her advice to plan my diet.”*

*(Participant 35)*

*Portion counting*

*“I think the most useful thing is portion counting… and how to choose food… I think it is more important to know the portion than a fixed diet plan… because you need to know clearly… I can only eat 3 pieces of meats, 2 bowls of vegetable in one meal… after I know the portion size, I won’t overeat.”*

*(Participant 103)*

*Healthier food choices*

*Nutritionist 2 reminded the participant the sequence of food intake when having hotpot, and when he needed to attend feast, he could eat the carbohydrate portion first before going there, then eat 1 piece of food from each dish.*

*(Participant 118, field note of diet consultation)*

*Strategies used to handle barriers*

The two major barriers reported were food craving and hunger. To overcome food craving, a limited portion was given to participants occasionally. More portions were allowed in the weight maintenance phase, as the diet was less stringent. Moreover, RDs/nutritionists advised participants to find a healthier alternative to cope with the craving.

*For snacking, Nutritionist 2 suggested the participant replace chocolates, chips and ice cream with homemade ice bars and told her how to make ice bars.*

*(Participant 23, field note of diet consultation)*

Hunger was more common in the early weight loss phase when participants had just started dieting. RDs/nutritionists suggested that participants could eat healthy snacks with low calories such as apples, celery and cherry tomatoes to satisfy the hunger.

*“Hunger… my nutritionist would encourage me: you can try to eat apple, or soda biscuit or those low-fat biscuits. That means… I have choices! I can choose something to fight hunger.”*

*(Participant 103)*

##### PA Knowledge

The findings of both interviews and observation suggest that all participants received PA knowledge from either RDs/nutritionists or the fitness specialist but some of them forgot what they had learned from the fitness specialist. In particular, one participant commented that she had attended only one PA consultation and there were too many postures to remember.

*“As there is only one [consultation]… I can’t remember clearly…There were too many postures… I can’t remember all of them. But I think they are similar.”*

*(Participant 104)*

Type of PA was the most common type of knowledge mentioned. Aerobic exercise was recommended for weight loss in both diet and PA consultations. The most common types of aerobic exercise were jogging, power walking and Skywalk. If aerobic exercise was not feasible for the participants, RDs/nutritionists recommended walking for at least 30 min per day to reduce sedentary time. Muscle strengthening exercises and stretching exercises were commonly recommended by the fitness specialist.

*“The fitness specialist taught me how to do that exercise…he asked me to do a power walk first… I followed his advice…then my nutritionist told me…what kind of exercise was good for firming specific parts of the body… I also followed her advice.”*

*(Participant 100)*

It was observed that adaption of PA into daily life and using heart rate as indicator of PA intensity were common examples of strategies to facilitate PA adherence. However, only those with high PA adherence mentioned adopting these strategies.

*Participant 84 told Nutritionist 2 that she usually did the step machine for 1 h after work. Nutritionist 2 recommended her to do the Skywalk, but the client said she found that she sweated more with the step machine than with the Skywalk. Nutritionist 2 asked for her heart rate during exercise. Participants 84 said it was about 160 rate per minute. Nutritionist 2 commented that was too rigorous for her.*

*(Participant 84, field note of diet consultation)*

#### 3.2.2. Theme 2: RD/Nutritionists or Fitness Specialist Enhanced Participants’ Self-Efficacy

##### Diet Self-Efficacy

Most participants perceived that they had more confidence in eating a healthy diet after joining CNSLMP. The major sources of diet self-efficacy were either from the nutrition knowledge delivered by RDs/nutritionists or the weight loss achievement after following the diet plans.

*“I think the confidence comes from successful weight loss. If you work so hard to not eat... but the weight didn’t drop, for sure I would have no confidence. Don’t you agree? I think because I had lost weight (Interviewer: So the confidence comes from weight loss?) Yes, I gained lots of confidence. If I can’t lose weight with a strict diet, I won’t follow it.”*

*(Participant 161)*

Analysis of field notes suggests two popular strategies being used by RDs/nutritionists to enhance diet self-efficacy during diet consultations: Complimenting and reinforcing past success. Complimenting refers to RDs/nutritionists praising participants for the good efforts they have made to follow diet plans. Reinforcing past success refers to RDs/nutritionists reminding participants of their past successful attempts of following diet plans.

*Participant had weight rebound and worried she could not adhere to diet plans after not following diet plans for a while. Nutritionist 6 reassured her that she could do it as she could start the diet plan gradually and she had succeeded before.*

*(Participant 104, field note of diet consultation)*

##### PA Self-Efficacy

Majority of the participants perceived their PA self-efficacy increased during the program. For those participants with a PA habit before joining CNSLMP, their PA self-efficacy either maintained or increased.

*“I had more confidence [in doing physical activity]. Now I keep the routine of playing basketball every Tuesday.”*

*(Participant 118)*

*“Interviewer: Did you gain more confidence in doing physical activity after joining?*

*Participant 104: No… I go dancing just because it’s my hobby.”*

In two of the diet consultations, RDs/nutritionists used praising as a strategy to enhance participants’ PA self-efficacy.

*Participant told Nutritionist 5 that he walked 20,000–30,000 steps during travel. Nutritionist 5 praised him that it’s good to walk a lot.*

*(Participant 103, field note of diet consultation)*

The findings of PA observation enhanced our understanding of the strategies that the fitness specialist used to enhance participants’ PA self-efficacy. The first strategy was demonstration of correct postures of PA, in particular for more complicated PA, such as Skywalk, power walk, high-intensity interval training (HIIT), stretching and muscle strengthening exercises. This is important, as incorrect postures not only hamper adherence but also pose risk of injury. The second strategy was to ask participants to try the postures during consultations (return-demonstration) so that the fitness specialist can correct their inappropriate postures on the spot.

#### 3.2.3. Theme 3: CNSLMP Did Not Affect Participants’ Motivation

##### Diet Motivation

Most participants perceived their motivation did not change much during the program and they already had high motivation before joining the program. The most frequently mentioned source of motivation was health, followed by body shape concern, family, and friends. A few participants mentioned that they were motivated to eat a healthy diet as they paid for the program and did not want to waste the money.

*“I think it’s because…I regained a lot of weight. And I did a body check around that time. I know some of the indicators were not good. The doctor also recommended me to lose weight.”*

*(Participant 35)*

*“I have a few reasons to follow this program. First, I have paid the fee. I don’t want to waste money. [laugh]. Second, you have to see a nutritionist… She will have put in so much effort in vain… [if] you don’t listen… I think it’s also very discouraging to her. Third, I have to be responsible for myself. Yes! All these drove me to… also your friends know you have joined this program [laugh]. If they found out you didn’t succeed, they would say something like “You see, you are still fat, I told you not to join.”*

*(Participant 254)*

##### PA Motivation

Only a few of them perceived CNSLMP to have enhanced their motivation. Perceived health and weight loss benefits were the major sources of motivation to do PA as reported by participants. However, many of them had these motivations before joining the program.

*“I always think doing exercise is one of the key factors to lose weight…therefore… if I have time, I will do it…”*

*(Participant 254)*

A few participants mentioned negative sources of motivation for PA. They were relying on diet to lose weight, tiredness after work, failing to lose weight through PA and no exercise buddy.

*“I thought meeting nutritionists would only focus on diet… I never thought I had to supplement with exercise…because I am afraid of doing exercise.”*

*(Participant 112)*

During consultations, RD/Nutritionists and the fitness specialist tried to motivate participants to do PA by stressing that PA is important for weight loss and weight maintenance. In addition, the fitness specialist also emphasized that muscle strengthening exercises are important for building up muscles and stretching exercises are important for relaxing muscles.

*Nutritionist 2 emphasized that PA is important for successful weight maintenance. She shared an example that one of her clients experienced weight rebound immediately after she stopped doing exercise.*

*(Participant 257, field note of diet consultation)*

## 4. Discussion

To our knowledge, this is the first study to explore the psychological predictors of dietary and PA adherence in an LMP among Chinese adults during the weight maintenance phase. It is found that participants had lower dietary adherence but similar PA adherence when compared to the adherence outcomes reported in the early weight loss phase [13]. Similarly, the failure of maintaining dietary changes is common in the existing literature [30,31,32,33].

A sequential explanatory strategy of the mixed methods approach, in which the analyses of quantitative data and qualitative data were conducted separately and the findings were integrated during interpretation stage, was adopted. The qualitative findings and quantitative findings were mostly supplementary to each other. The findings of the quantitative study partially supported the study hypotheses that different psychological predictors were identified for diet and PA adherence. In the following paragraphs, we discussed the four psychological factors investigated in this study based on the quantitative and qualitative findings.

### 4.1. Stage of Change

Regardless of diet or PA behaviors, increase in stage of change, which was analyzed as a dichotomized variable, was found to be an independent predictor of adherence at 10-month follow-up. This significant finding supported our postulation that the insignificant finding regarding the relationship between stage of change and adherence observed in the weight loss phase was due to insufficient time allowed for stage of change to proceed [13]. In addition, being in the “action” stage of change at baseline was significant in the multivariable models, implying both pre-treatment and increase in stage of change were important for better adherence during weight maintenance phase. Nevertheless, the longitudinal relationship between stage of change and dietary or PA behavioral changes has seldom been examined in the existing literature. In a recent systematic review on lifestyle interventions using the stage of change model, the authors concluded that there has been paucity of evidence on the effectiveness of such interventions on long term improvement in eating habits and PA [34].

### 4.2. Knowledge

For nutrition knowledge, both baseline and increase in nutrition knowledge independently predicted better dietary adherence at 10 months, implying that pre-treatment nutrition knowledge is equally important as an increase in nutrition knowledge for promoting a better adherence to weight maintenance diets. The qualitative study findings suggest that nutrition knowledge was gained through CNSLMP. During interviews, participants reported that they gained a variety of nutrition knowledge from RDs/nutritionists. Moreover, it was observed that the majority of the time was spent on delivering nutrition knowledge during diet consultations. In addition, the four main types of knowledge (healthier food choices, portion counting, strategies used to handle barriers and individualized diet plans) identified in the qualitative study were key knowledge components of CNSLMP. Notably, the interviews were conducted on average 11 months post weight loss phase, implying that nutrition knowledge retention was good. Similarly, nutrient knowledge retention was reported by participants of an 18-week group-based comparative trial that promoted weight loss using an energy-restricted diet and the portion control knowledge was identified as the key facilitator to weight maintenance during focus group interviews [35]. In view of the scarce evidence on the longitudinal relationship between nutrition knowledge and dietary behavior, our findings add knowledge to existing research.

Previous cross-sectional studies on Hong Kong Chinese adults suggested a positive relationship between knowledge and levels of PA [27,36]. However, this study found that increase in PA knowledge was not a significant predictor of PA adherence. The insignificant finding could be explained by the finding of the qualitative study that knowledge retention was poor among participants and most of them did not apply the strategies recommended by the fitness specialist. Another possible reason could be the less frequent PA consultation offered in CNSLMP.

### 4.3. Self-Efficacy

There has been some evidence supporting the longitudinal relationship between self-efficacy and weight maintenance [37,38,39]. In this study, increase in PA self-efficacy predicted PA adherence but it is somewhat surprising that no significant relationship was noted between diet self-efficacy and dietary adherence in the quantitative study. However, the majority of the interview participants perceived their diet self-efficacy to be enhanced by RDs/nutritionists through increase in nutrition knowledge and successful weight loss after following the diet plans prescribed by RDs/nutritionists. Additionally, the finding from diet observation on the strategies that RDs/nutritionists used to enhance participants’ diet self-efficacy: compliments and reinforcement of pass success, complemented the findings from interviews. 

Regarding PA self-efficacy, the qualitative study finding that the majority of the interview participants perceived their PA self-efficacy increased during the program concurs with the quantitative studying finding that there was a significant increase in PA self-efficacy at 10 months. Furthermore, the finding from PA observation that the fitness specialist attempted to enhance participants’ PA self-efficacy through demonstration and re-turn demonstration complemented the findings from interviews. These strategies are examples of “vicarious experience” and “enactive attainment” suggested by Bandura to enhance self-efficacy [40].

### 4.4. Motivation

The relationship between motivation and weight maintenance is less well established in the literature [3]. In the present study, neither PA nor diet motivations were predictors of adherence. This result may be explained by the fact that participants had high baseline motivation, which might have limited the room for further improvement. The qualitative findings indicated that most participants perceived their motivation not to be affected by the program and the major source of motivation was health concern. According to Self-Determined Theory, health concern can be classified as autonomous motivation [41,42]. Notably, the health concern mentioned for diet motivation was mostly disease specific but not for PA motivation. In other words, participants were motivated to eat a healthy diet for disease management or prevention. Another possible explanation for the insignificant relationship between PA motivation and PA adherence could be that the program was not effective in addressing the negative sources of motivation including relying on diet to lose weight, tiredness after work, failing to lose weight through PA and no exercise buddy. In this regard, we did not identify any strategies that had been used to address negative sources of motivation in the qualitative study. Therefore, more effort should be made to reduce the influence of negative sources of PA motivation.

### 4.5. Strengths and Limitations

There are several noteworthy strengths in this study. First, our study is one of the first studies to investigate the longitudinal relationship of psychosocial factors with dietary and PA adherence in lifestyle modification program for weight management. Second, the use of a mixed methods strategy, combining both quantitative and qualitative analyses, offers a more comprehensive understanding of the findings than either quantitative or qualitative analysis alone. Third, the study was conducted in a real-world setting with minimal experimental controls. The participants in this study represented a population who were actively seeking weight loss. Our findings could be translated into practice in order to actually impact public health. However, interpretation of study findings is limited by the high dropout rate, the short duration of the weight maintenance phase, the over-representation of highly motivated women, the adoption of convenience sampling, the use of self-report measures, the observational phase not being illustrative of the weight loss phase, and the presence of other factors that may affect adherence, such as biological factors [43]. As we purposely recruited all eligible clients of CNSLMP, the low percentage of men enrolled in our study reflected the fact that the majority of CNSLMP clients were women.

### 4.6. Implications

There are several implications that may be drawn from this study. First, it is important to assess nutrition knowledge and stage of change at the beginning of weight loss programs to identify those who are less likely to adhere to long-term lifestyle changes. Second, RDs/nutritionists should provide tailor-made nutrition knowledge and apply stage of change concepts during the early weight maintenance phase. Four types of knowledge should be considered: individualized diet plans, portion counting, healthier food choices and strategies to handle barriers. Third, adopting strategies to improve PA self-efficacy and applying stage of change concept are recommended for highly motivated participants in early weight maintenance phase. Demonstration and return demonstration were examples of effective strategies to improve PA self-efficacy. Fourth, the findings support the use of program-specific dietary and PA adherence scores as indicators of dietary and PA adherence.

## 5. Conclusions

Given the limited evidence exploring the relationship between psychological factors and adherence to lifestyle changes, the findings of this study add knowledge to the existing literature and provide evidence for the development of sustainable obesity management strategies. Furthermore, the findings support the use of program-specific dietary and PA adherence scores as indicators of adherence to LMP. Future studies should include longer term follow-ups and develop strategies to recruit less motivated male participants.

## Figures and Tables

**Table 1:** Comparison of baseline characteristics between completers and non-completers of the study.

	Completers (*N* = 140)	Non-Completers (*N* = 65)	*t* or *χ*^2^
	Mean ± SD/*N* (%)	Mean ± SD/*N* (%)	
Age (years)	39.2 ± 10.5	38.1 ± 10.3	−0.718
BMI (kg/m^2^)	28.0 ± 4.0	29.1 ± 4.5	1.723
Gender			5.959 *
Female	116 (82.9%)	44 (67.7%)	
Male	24 (17.1%)	21 (32.3%)	
Marital status			0.208
Married	62 (44.3%)	31 (47.7%)	
Never married/Divorced/Widow	78 (55.7%)	34 (52.3%)	
Level of education			0.013
Tertiary or above	83 (59.3%)	38 (58.5%)	
Below tertiary	57 (40.7%)	27 (41.5%)	
Occupation ^!^			1.008
Managers and administrators	31(22.3%)	12 (18.8%)	
Professionals or associate professionals	41 (29.5%)	23 (35.9%)	
Clerical or services workers	37 (26.6%)	17 (26.6%)	
Others ^#^	30 (21.6%)	12 (18.8%)	
Working pattern			0.391
Full time	115 (82.1%)	51 (78.5%)	
Not Full Time	25 (17.9%)	14 (21.5%)	
Monthly family income (HKD) ^!^			2.332
≤30,000	42 (31.1%)	26 (40.0%)	
30,001–60,000	58 (43.0%)	21 (32.3%)	
>60,000	35 (26.0%)	18 (27.7%)	
Number of chronic diseases			2.730
None	84 (60.0%)	31 (47.7%)	
1 or more	56 (40.0%)	34 (52.3%)	
Current drinking habit			0.053
No	97 (69.3%)	44 (67.7%)	
Yes	43 (30.7%)	21 (32.3%)	
Current smoking habit			1.484
No	121(86.4%)	60 (92.3%)	
Yes	19 (13.6%)	5 (7.7%)	
Weight change during past 10 years			0.231
Gradually increased	91 (65.0%)	40 (61.5%)	
Ups and downs/No big changes	49 (35.0%)	25 (38.5%)	
Number of previous weight loss attempts			2.292
None	23 (16.4%)	15 (23.1%)	
1	39 (27.9%)	20 (30.8%)	
2	34 (24.3%)	15 (23.1%)	
3 or more	44 (31.4%)	15 (23.1%)	
Center location			0.212
Business district	32 (22.9%)	13 (20.0%)	
Residential area	108 (77.1%)	52 (80.0%)	
Joined CNSLMP before			9.603 **
No	113 (80.7%)	63 (97.0%)	
Yes	27 (19.3%)	2 (3.0%)	

*N* = 205; SD: Standard Deviation; BMI: Body Mass Index. * *p* < 0.05; ** *p* < 0.01. **^!^** different sample sizes due to missing data. ^#^ Others included unemployed (*N* = 2), housewife (*N* = 7), student (*N* = 9), retired (*N* = 9), elementary occupations (*N* = 1), skilled agricultural and fishery worker (*N* = 1), self-employed (*N* = 10) and church workers (*N* = 3).

**Table 2:** Comparison of psychological outcomes at baseline and 10-month follow-up.

Outcomes [Range]	Baseline	10 Months	t/z
	Mean ± SD/Median (IQR)	Mean ± SD/Median (IQR)	
Nutrition knowledge score [0–22]	10.72 ± 3.12	13.95 ± 2.74	13.14 ***
PA knowledge score [0–11]	7.26 ± 1.39	7.75 ± 1.40	3.51 ***
Diet self-efficacy [0–28] (SRAHP- Nutrition subscale)	21.36 ± 3.48	22.89 ± 3.08	6.03 ***
PA self-efficacy [0–28] (SRAHP- PA subscale)	16.91 ± 5.07	19.05 ± 4.73	5.08 ***
Autonomous motivation for healthy diet [1–42] (TSRQ-autonomous subscale)	34.72 ± 5.12	35.03 ± 5.14	0.74
Autonomous motivation for PA [1–42] (TSRQ-autonomous subscale)	33.29 ± 5.82	33.02 ± 6.36	−0.55
Controlled motivation for healthy diet [1–42] (TSRQ-controlled subscale)	24.15 ± 6.19	25.37 ± 6.26	2.62 **
Controlled motivation for PA [1–42] (TSRQ-controlled subscale)	22.42 ± 6.36	23.74 ± 7.15	2.51 *
^!^ Diet motivation to improve body image [1–7]	6 (5 to 7)	6 (5 to 7)	−1.23
^!^ PA motivation to improve body image [1–7]	6 (5 to 7)	6 (5 to 7)	−1.19
^^^ Stage of change for healthy diet [1–5]	3 (2 to 3)	4 (3 to 5)	8.75 ***
^^^ Stage of change for PA [1–5]	3 (2 to 4)	3 (3 to 5)	4.37 ***

*N* = 140; SD: Standard Deviation; IQR: Interquartile range; PA: Physical Activity; SRAHP: Self Rated Abilities for Health Practices Scale; TSRQ: Treatment Self-Regulation Questionnaire; * *p* < 0.05; ** *p* < 0.01; *** *p* < 0.001. ^!^ *N* = 139, different sample sizes due to missing data. ^^^ stage of change: 5: maintenance, 4: action, 3: determination, 2: contemplation, 1: pre-contemplation.

**Table 3:** Results of univariate and multivariable regression analyses for dietary adherence score.

	Univariate Model		Multivariable Regression Model ^!^	
	*β*	95%CI	*β*	95%CI
Changes in psychological variables				
Nutrition knowledge score	0.32	[−0.03, 0.66]	0.57 **	[0.16, 0.98]
Diet self-efficacy (SRAHP-Nutrition subscale)	0.02	[−0.31, 0.36]	−0.09	[−0.50, 0.32]
Autonomous motivation for healthy diet (TSRQ-autonomous subscale)	0.16	[−0.04, 0.37]	0.10	[−0.13, 0.33]
Controlled motivation for healthy diet (TSRQ-controlled subscale)	−0.07	[−0.25, 0.12]	−0.09	[−0.29, 0.10]
^$,!^ Diet motivation to improve body image (Ref: No change/decreased)				
Low to high	−1.23	[−4.89, 2.40]	−1.14	[−4.87, 2.59]
Stage of change for healthy diet (Ref: +1 stage or below)				
+2 stages or more	2.16 *	[0.15, 4.17]	2.59 *	[0.42, 4.76]
Baseline psychological variables				
Nutrition knowledge score	0.18	[−0.14, 0.51]	0.50 *	[0.12, 0.87]
Diet self-efficacy (SRAHP-Nutrition subscale)	0.22	[−0.07, 0.51]	−0.04	[−0.40, 0.32]
Autonomous motivation for healthy diet (TSRQ-autonomous subscale)	−0.10	[−0.30, 0.10]	0.06	[−0.17, 0.29]
Controlled motivation for healthy diet (TSRQ-controlled subscale)	0.00	[−0.16, 0.16]	−0.02	[−0.21, 0.16]
^$,!^ Diet motivation to improve body image (Ref: low)				
High	−2.27 *	[−4.40, −0.15]	−2.07	[−4.37, 0.24]
Stage of change for healthy diet (Ref: Contemplation/Pre-contemplation)				
Determination	−0.48	[−2.67, 1.71]	0.15	[−1.91, 2.21]
Action/Maintenance	2.34	[−0.62, 5.31]	3.95 *	[0.88, 7.03]
Covariates				
Married (Ref: Not married)	1.67	[−0.36, 3.69]	2.41 *	[0.33, 4.49]
Full time work (Ref: Not full time)	−4.30 **	[−6.85, −1.75]	−3.19 *	[−5.67, −0.72]
Eating out frequency at 10 months (Ref: ≤3 times a week)				
4–6 times a week	−2.88 *	[−5.38, −0.38]	−1.96	[−4.34, 0.42]
At least once a day	−2.35	[−4.78, 0.70]	−1.22	[−3.54, 1.10]
Jointed CNSLMP before (Ref: No)	1.96	[−0.60, 4.51]	2.11	[−0.31, 4.53]
Total number of nutrition consultations attended (Ref: <10)				
10−16	2.60 *	[0.24, 4.96]	2.63 *	[0.42, 4.84]
17−34	3.52 **	[1.08, 5.97]	2.48 *	[0.08, 4.87]
*R^2^*			0.36	
*F*			3.30 ***	

*N* = 140. CI: Confidence Interval; SRAHP: Self Rated Abilities for Health Practices Scale; TSRQ: Treatment Self-Regulation Questionnaire; ^$^ Diet motivation to improve body image score: Low < 6, High ≥ 6; ^!^ *N* = 139, different sample sizes due to missing data. * *p* < 0.05; ** *p* < 0.01, *** *p* < 0.001.

**Table 4:** Results of univariate and multivariable regression analyses for PA adherence score.

	Univariate Model		Multivariable Regression Model ^!^	
	*β*	95%CI	*β*	95%CI
Changes in psychological variables				
PA knowledge score	−0.02	[−0.24, 0.20]	−0.09	[−0.32, 0.14]
PA self-efficacy (SRAHP-PA subscale)	0.16 ***	[0.09, 0.22]	0.16 ***	[0.07, 0.24]
Autonomous motivation for PA (TSRQ-autonomous subscale)	0.08 **	[0.02, 0.15]	0.04	[−0.03, 0.11]
Controlled motivation for PA (TSRQ-controlled subscale)	−0.00	[−0.06, 0.06]	−0.01	[−0.07, 0.04]
^$^ PA motivation to improve body image (Ref: No change/decreased)				
Low to high	0.62	[−0.54, 1.77]	1.08	[−0.15, 2.32]
Stage of change for PA				
(Ref: No change/decreased)				
Increased	0.97 **	[0.27, 1.67]	0.83 *	[0.07, 1.58]
Baseline psychological variables				
PA knowledge score	−0.09	[−0.35, 0.17]	−0.08	[−0.37, 0.20]
PA self-efficacy (SRAHP-PA subscale)	0.01	[−0.07, 0.08]	0.06	[−0.03, 0.14]
Autonomous motivation for PA (TSRQ-autonomous subscale)	0.02	[−0.04, 0.09]	0.00	[−0.07, 0.07]
Controlled motivation for PA (TSRQ-controlled subscale)	0.03	[−0.03, 0.08]	0.03	[−0.03, 0.08]
^$,!^ PA motivation to improve body image (Ref: low)				
High	−0.23	[−0.99, 0.54]	0.40	[−0.41, 1.21]
Stage of change for PA (Ref: Contemplation/Pre-contemplation)				
Determination	−0.33	[−1.15, 0.49]	0.06	[−0.75, 0.86]
Action/Maintenance	0.77	[−0.17, 1.71]	1.13 *	[0.11, 2.15]
Covariates				
Current drinking habit (Ref: No)	0.68	[−0.95, 1.44]	0.45	[−0.27, 1.18]
Full time work (Ref: Not full time)	−0.67	[−1.60, 0.26]	−0.33	[−1.21, 0.55]
Jointed CNSLMP before (Ref: No)	−0.99 *	[−1.88, −0.10]	−0.73	[−1.53, 0.08]
Had PA consultation (Ref: No)	0.86 *	[0.06, 1.65]	0.56	[−0.16, 1.29]
Total no. of CNSLMP sections attended (Ref: <10)				
10–16	−0.09	[−0.92, 0.74]	−0.27	[−1.03, 0.48]
≥17	1.14 *	[0.27, 2.00]	0.64	[−0.18, 1.45]
*R^2^*			0.38	
*F*			3.78 ***	

*N* = 140; CI: Confidence Interval; PA: Physical Activity; SRAHP: Self Rated Abilities for Health Practices Scale; TSRQ: Treatment Self-Regulation Questionnaire; ^$^ PA motivation to improve body image score: Low < 6, High ≥ 6; **^!^** *N* = 139, different sample sizes due to missing data. * *p* < 0.05; ** *p* < 0.01; *** *p* < 0.001.

**Table 5:** Baseline demographic characteristics and program experience of interview participants (n = 26).

	*N* (%)
Baseline characteristics	
Age (years) ^a^	
<30	4 (15.4%)
30–39	10 (38.5%)
40–50	4 (15.4%)
50–65	8 (30.8%)
BMI (kg/m^2^) ^a^	
23.0–24.9	3 (11.5%)
25.0–29.9	18 (69.2%)
≥30	5 (19.2%)
Gender	
Female	23 (88.5%)
Male	3 (11.5%)
Marital Status	
Married	12 (46.2%)
Never married/Divorced/Widow	14 (53.8%)
Level of Education	
Below tertiary	9 (34.6%)
Tertiary or above	17 (65.4%)
Occupation	
Managers and administrators	3 (11.5%)
Professionals or associate professionals	11 (42.3%)
Clerical or services workers	8 (30.8%)
Others	4 (15.4%)
Working Pattern	
Full time	22 (84.6%)
Not Full Time	4 (15.4%)
Monthly Family Income (HKD)	
≤30,000	10 (38.5%)
30,001–60,000	11 (42.3%)
>60,000	5 (19.2%)
No. of chronic diseases	
None	21 (80.8%)
1 or more	5 (19.2%)
Number of previous weight loss attempt ^a^	
None	2 (7.7%)
1	10 (38.5%)
2	8 (30.8%)
3 or more	6 (23.1%)
CNSLMP experience	
Center location	
Business district	4 (15.4%)
Residential area	22 (84.6%)
Joined CNSLMP before	
No	20 (76.9%)
Yes	6 (23.1%)
Total number of nutrition consultations attended	
<10	8 (30.8%)
10–16	9 (34.6%)
17–33	9 (34.6%)
Total number of PA consultations	
None	5 (19.2%)
1	15 (57.7%)
2	6 (23.1%)
Weight loss phase duration	
<4 months	7 (26.9%)
4–7.9 months	10 (38.5%)
8–13 months	9 (34.6%)
Weight maintainer ^b^	
No	5 (19.2%)
Yes	21 (80.8%)

BMI: Body Mass Index; PA: Physical Activity. ^a^ Total percentage does not add up to exactly 100% due to round off error. ^b^ Definition: <5% Weight change since last weight loss appointment.

**Table 6:** Summary of the themes, subthemes and codes.

Themes	Subthemes	Codes
RDs/nutritionists or fitness specialist enhanced participants’ knowledge.	Nutrition knowledge	Individualized diet plans Portion counting Healthier food choices Strategies used to handle barriers
	PA knowledge	Type of PA Strategies used to facilitate adherence
2. RDs/nutritionists or fitness specialist enhanced participants’ self-efficacy.	Diet self-efficacy	Perceived change in self-efficacy Sources of self-efficacy Strategies used to enhance self-efficacy
	PA self-efficacy	Perceived change in self-efficacy Sources of self-efficacy Strategies used to enhance self-efficacy
3. CNSLMP did not affect participants’ motivation	Diet motivation	Perceived change in motivation Sources of motivation
	PA motivation	Perceived change in motivation Sources of motivation

CNSLMP: Lifestyle Modification Program in the Center for Nutritional Studies; RD: Registered Dietitians; PA: Physical activity.

## Supplementary Materials

The following are available online at https://www.mdpi.com/2072-6643/12/5/1379/s1, Table S1: Interview guide for the qualitative study.
