# Supplementary material for: Psychological Factors of Long-Term Dietary and Physical Activity Adherence among Chinese Adults with Overweight and Obesity in a Community-Based Lifestyle Modification Program: A Mixed-Method Study
Source: Nutrients. 2020 May 12;12(5):1379. doi: 10.3390/nu12051379 (PMC7284498; doi:10.3390/nu12051379)
Supplement: Supplementary file 1 [file nutrients-12-01379-s001.zip › Table S1. Interview guide.doc]

Table S1. Interview guide for the qualitative study

1. Please describe your overall experience in CNSLMP and the lifestyle changes (especially diet and PA) upon completion of program.
2. How do you perceive your adherence to CNSLMP (probe: diet and PA)?
   a. Upon completion of weight loss phase in CNSLMP, did you follow the diet maintenance advice? If yes, in what extent?
3. Knowledge
   a. Do you think CNSLMP help you improved nutrition knowledge (PA knowledge)? If yes, please elaborate.
   b. How did CNSLMP help you in this aspect?
   c. Did the knowledge help you follow the diet (PA) plan? If yes, please elaborate.
   d. Does the knowledge still affect you after CNSLMP? If yes, please elaborate.
4. Self-efficacy
   a. How do you perceive your confidence in maintaining a healthy diet (do regular PA) before CNSLMP?
   b. Do you think CNSLMP help you increase the confidence? If yes, what kind of confidence?
   c. How did CNSLMP help you in this aspect?
   d. Did the confidence help you follow the diet (PA) plan? If yes, please elaborate.
   e. Does the confidence still affect you after CNSLMP? If yes, please elaborate.
5. Motivation
   a. How do you perceive your motivation to maintain a healthy diet (do regular PA) before joining CNSLMP? Why?
   b. Do you think CNSLMP affect your motivation? Why?
   c. Did the motivation help you follow the diet (PA) plan? If yes, please elaborate.
   d. Does the motivation still affect you after CNSLMP? If yes, please elaborate.
6. Are there any other barriers to prevent you from following the diet (PA) plan before and after CNSLMP? If yes, please elaborate.
7. Are there any other factors that you think is important to your adherence to CNSLMP? If yes, what are they?

Thank you for your valuable opinions!
